# Time Interval From Early-Term Antenatal Corticosteroids Administration to Delivery and the Impact on Neonatal Outcomes

**DOI:** 10.3389/fped.2022.836220

**Published:** 2022-04-11

**Authors:** Jing Li, Jing Zhang, Qingfei Hao, Yanna Du, Jie Lu, Haoming Chen, Xiuyong Cheng

**Affiliations:** Department of Neonatology, The First Affiliated Hospital of Zheng Zhou University, Zhengzhou, China

**Keywords:** antenatal corticosteroids, gestational diabetes mellitus, neonatal respiratory distress, early term scheduled cesarean section, hypoglycemia

## Abstract

**Objectives:**

To determine the association between the time interval from antenatal corticosteroids administration to delivery and neonatal complications in diabetic mothers undergoing early term (37+0 to 38+6 weeks) scheduled cesarean section (ETSCS).

**Study Design:**

A retrospective cohort study of women with any form of diabetes in pregnancy undergoing ETSCS was included. Cases were stratified into the following groups based on the time interval from the first dose of corticosteroids administration to delivery: <2, 2–7, and >7 days. Women undergoing ETSCS, who did not receive corticosteroids were included as controls. We assessed the association between the time interval and neonatal outcomes in a multivariate regression model that controlled for potential confounders. Primary outcomes were the incidence of respiratory distress syndrome (RDS)/transient tachypnea of the newborn (TTN) and neonatal hypoglycemia.

**Results:**

The study cohort comprised 1,165 neonates. Of those, 159 (13.6%) were delivered within 2 days of maternal corticosteroids administration, 131 (11.2%) were delivered within 2–7 days after maternal corticosteroids administration, and 137 (11.8%) delivered more than 7 days after maternal corticosteroids administration. The remaining 738 (63.3%) were not exposed to corticosteroids. Multivariate analysis demonstrated that delivery within any time of antenatal corticosteroids administration was not associated with decreased risks of RDS/TTN. The risk of neonatal hypoglycemia was highest in the delivery of <2 days group (adjusted odds ratio [aOR]: 2.684, 95% confidence interval [CI]: 1.647–4.374 for control group; aOR: 2.827, 95% CI: 1.250–6.392 for delivery 2–7 days group; aOR:2.975, 95% CI: 1.265–6.996 for delivery >7 days group).

**Conclusions:**

Corticosteroids treatment for diabetic mothers undergoing ETSCS was not associated with beneficial neonatal respiratory outcomes. In addition, delivery, <2 days after antenatal corticosteroids administration was associated with an increased risk of neonatal hypoglycemia.

## Introduction

Infants born by elective cesarean delivery at term are at increased risk for developing respiratory complications compared with those born by vaginal delivery, particularly if born *via* cesarean section (CS) at 37+0 to 38+6 weeks of gestation (1–3). Gestational diabetes mellitus (GDM) is a common complication of pregnancy, and the prevalence of GDM in mainland China is 14.8%, with an increasing trend in recent years (4). Neonates to diabetic mothers are also subjected to a higher risk of respiratory morbidity than neonates of non-diabetic mothers (5–8). Such situations raise the question of the place of antenatal corticosteroids (ACS) in pregnant women with GDM or diabetes in pregnancy (DIP) undergoing an early term scheduled cesarean section (ETSCS) (9).

In pregnancies uncomplicated by diabetes, ACS treatment before term elective CS reduces the incidence of respiratory distress syndrome (RDS) and transient tachypnea of the newborn (TTN) and has been proposed as a way of reducing adverse neonatal outcomes in this population (10–12). Whereas, there appears to be insufficient evidence regarding the benefit of ACS in the early term period in pregnancies complicated by diabetes, it is because women with diabetes have largely been excluded (13), remained unidentified (11), or were included only in small numbers (6/819 women with GDM) (10) in studies investigating the efficacy of ACS. Recently, two retrospective studies investigating the efficacy and safety of ACS in women with GDM undergoing a term CS obtained inconsistent findings (14, 15). The time interval between corticosteroids administration and CS in one study ranged from 1 to 46 days (14), which may dilute the benefit of ACS treatment given that the optimal intervention timing is when delivery occurs within 2–7 days of administration (16). The corticosteroids administration timing was not specified in the other study, which found a reduction of respiratory complications (15). It is unclear whether exposure to ACS at different time points for these early-term infants is beneficial or may lead to some harm. The aim of our study, therefore, was to investigate whether the time interval from early-term corticosteroids administration to delivery is associated with a change in the likelihood of RDS/TTN, and neonatal hypoglycemia.

## Materials and Methods

We conducted a retrospective cohort study of all women who were diagnosed with GDM, DIP, or pre-existing DIP undergoing an early term (37^+0^ to 38^+6^) elective cesarean section in the First Affiliated Hospital of Zhengzhou University, Zhengzhou, China, between 2016 and 2021. Exclusion criteria included multiple gestations, major fetal anomaly or fetal chromosomal abnormalities, and women who underwent an emergency CS. Cases were stratified into the following 4 groups based on the time interval from the first dose of antenatal corticosteroid administration to delivery: <2, 2–7, >7 days, and control group (corticosteroids not administered).

The following demographic and obstetrical variables were recorded: type of diabetes, ways of glycemic control, maternal age ≥35 years, hypertensive disorders, body mass index (BMI), indications to initiate treatment with ACS, and glycated hemoglobin (HbA1c) in the second or third trimester. The neonatal baseline data collected included the following: gestational age, birth weight, small/large for gestational age, male sex, and 1-min Apgar score <7. The primary outcomes of interest were RDS/TTN and neonatal hypoglycemia. Secondary outcomes included neonatal intensive care unit (NICU) admissions and length of stay in NICU.

The GDM or DIP is diagnosed based on one or more abnormal glucose values: (GDM: fasting blood glucose ≥5.1 mmol/L; 1-h post 75 g OGTT≥10 mmol/L; and 2-h post 75 g OGTT≥8.5 mmol/L, DIP: fasting glucose ≥7 mmol/L, 2-h glucose ≥11.1 mmol/L, following World Health Organization criteria) (17). The diagnosis of emergency cesarean delivery referred to the literature (18). The birth weight percentile was calculated using the Fenton curves in 2013 (19). Diagnostic criteria for RDS include the following: (1) representative clinical manifestations, including progressive respiratory distress occurring shortly after birth, tachypnea, flaring of the nostrils, expiratory grunting, cyanosis, positive three-concave sign, reduced or absent breathing sounds, and severe dyspnea, require continuous positive pressure ventilatory support for at least 72 h; (2) typical chest X-ray findings, such as fine granular densities, ground-glass opacity, air bronchogram sign, or white lungs; (3) arterial blood gas analysis showing hypoxia, hypercapnia, and oxygen tension/fraction of inspired oxygen ratio (PaO_2_/FiO_2_) ≤200 mmHg (20). The chest X-ray findings of TTN are mainly interstitial, alveolar, and interlobular pleural effusion. Neonatal hypoglycemia was defined as glucose levels ≤2.2 mmol/L within 24 h post-delivery (21). All neonates with a glucose level of ≤2.6 mmol/L were subsequently treated using a regimen that included intravenous dextrose therapy and surveillance in our unit.

Newborn exposure to ACS was defined as the administration of at least 1 of the 4 doses of 6 mg dexamethasone, given intramuscularly 12 h apart, as part of an antenatal corticosteroids course. The indications for ACS administration in this cohort were classified in a hierarchical classification system as one of the following: (1) preterm labor defined as regular uterine contractions causing cervical changes; (2) preterm pre-labor rupture of membranes (PPROM); (3) fetal indications (such as intrauterine growth restriction, oligohydramnios, or placental insufficiency); (4) maternal indications (such as hypertensive disorders, GDM, or DIP); (5) abnormal vaginal bleeding (such as placental abruption and placenta previa); (6) asymptomatic changes of the cervix (cervical length of <15 mm and/or dilation of >4 cm). However, the decision to administer ACS was ultimately left to the discretion of the obstetrician.

### Statistical Analysis

Data analysis was performed with the SPSS version 22.0 software. Continuous variables were compared among groups using the ANOVA test. The LSD test (for homogeneity of variance) or the Tamhane's test (heterogeneity of variance) was used for comparison between any two groups. The chi-square test with Bonferroni correction was used for categorical variables. Differences were considered significant when the *p*-value was < 0.05. Multivariable logistic regression analysis was performed to evaluate the association between the time interval and neonatal outcomes while adjusting for potential confounders.

## Results

**Figure 1:**
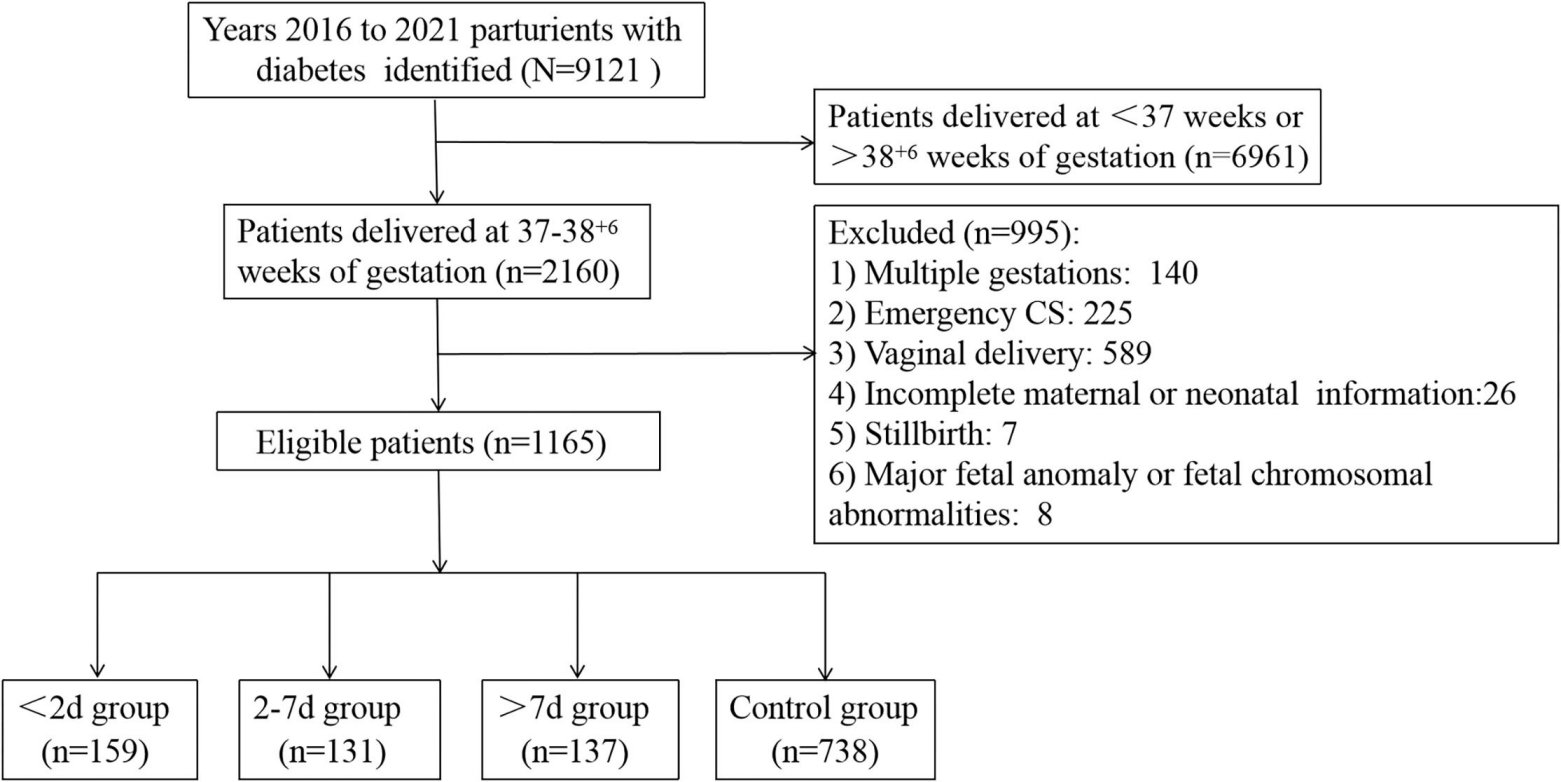
Flow chart of included patients.

A total of 9,121 parturients with GDM or DIP were identified during the study period, and 1,165 of them were included in the study (Figure 1). Of those, 159 (13.6%) delivered within 2 days of ACS administration, 131 (11.2%) delivered within 2–7 days after ACS administration, and 137 (11.8%) delivered more than 7 days after ACS administration. Characteristics of the included infants and their mothers are shown in Table 1. There were statistically significant differences in the baseline characteristics, such as maternal hypertensive disorders, doses of ACS treatment, gestational age at delivery, birth weight, and the percentage of small for gestational age among the 4 groups (Table 1). The proportion of maternal age ≥35 years, type of diabetes, ways of glycemic control, BMI, HbA1c in the second or third trimester, neonatal sex, and the proportion of 1-m Apgar score of <7 were all similar among the 4 groups (Table 1). Women with an ACS-delivery interval of <2 and 2–7 days were more likely to have received ACS for maternal indications rather than for fetal indications (Table 2).

**Table 1:** Characteristics of the studied groups.

**Variables**	**Delivered <2 d after ACS administration (*n =* 159)**	**Delivered 2–7 d after ACS administration (*n =* 131)**	**Delivered >7 d after ACS administration (*n =* 137)**	**ACS not administerd (*n =* 738)**	***P-value***
**Type of diabetes**, ***n*** **(%)**					0.337
GDM	152 (95.6)	120 (91.6)	132 (96.4)	696 (94.3)	
DIP	7 (4.4)	11 (8.4)	5 (3.6)	42 (5.7)	
**Ways of glycemic control**, ***n*** **(%)**					0.101^f^
Diet only	110 (69.6)	77 (59.7)	74 (54.4)^a^	477 (64.7)	
Insulin and diet	48 (30.4)	52 (40.3)	62 (45.6)^a^	258 (35.0)	
Medication and diet	0	0	0	2 (0.3)	
Maternal age≥35 years, *n* (%)	53 (33.3)	48 (36.6)	60 (43.8)	270 (36.6)	0.298
Hypertensive disorders, *n* (%)	16 (10.1)	14 (10.7)	26 (19.0)	73 (9.9)^**c**^	**0.019**
HbA1c in second or third trimester, %, median (Range), *n*	5.5 (4.9–6.9) 102	5.7 (4.5–9.3) 87	5.6 (2.7–7.6) 87	5.6 (1.7–8.4) 405	0.392
BMI (kg/m^2^), mean ± SD	28.6 ± 4.0	29.8 ± 5.0^a^	28.6 ± 5.0	28.9 ± 4.4	0.165
Full course of ACS, *n* (%)	50 (31.4)	125 (95.4)^a^	132 (97.1)^a^	0^b^^c^	**<0.001**
Gestational age, mean ± SD	37.8 ± 0.5	37.8 ± 0.5	38.0 ± 0.6^b^	38.3 ± 0.6^a^^b^^c^	**<0.001**
Birth weight (g), mean ± SD	3251.1 ± 477.2	3173.8 ± 521.7	3177.9 ± 500.2	3404.2 ± 486.9^a^^b^^c^	**<0.001**
Small for gestational age, *n* (%)	3 (1.9)	5 (3.8)	4 (2.9)	2 (0.3)^**abc**^	**0.001**
Large for gestational age, *n* (%)	2 (1.3)	2 (1.5)	2 (1.5)	10 (1.4)	1.000
Male sex, *n* (%)	91 (57.2)	79 (60.3)	71 (51.8)	424 (57.5)	0.541
1-min Apgar score <7, *n* (%)	1 (0.6)	1 (0.8)	3 (2.2)	4 (0.5)	0.514
RDS (including TTN), *n* (%)	4 (2.5)	0	2 (1.5)	14 (1.9)	0.154
Serum glucose level, median (Range)	2.5 (0.8–5.2)	2.8 (1.2–8.5)	2.7 (1.7–6.0)	2.8 (1.1–6.2)^a^	**0.024**
Neonatal hypoglycemia, n (%)	36 (22.6)	15 (11.5)	13 (9.5)^a^	62 (8.4)^a^	**<0.001**
Times of hypoglycemia, median (Range)	1 (1–3)	1 (1–2)	1 (1–1)^b^	1 (1–2)	**0.013**
NICU admission, *n* (%)	37 (23.3)	33 (25.2)	26 (19.0)	117 (15.9)^a^	**0.023**
Days in NICU, median (Range)	5.5 (1–23)	5.5 (2–24)	6 (1–22)	6 (1–38)	0.846

*PROM, premature rupture of membranes; ACS, antenatal corticosteroids. Bold indicate statistically signifcant*.

*Post-hoc method by Bonferroni*:

a *Means vs. Delivered <2 d after ACS administration group*.

b *Means vs. Delivered 2–7 d after ACS administration group*.

c *Means vs. Delivered >7 d after ACS administration group. Bold values are statistically significant*.

**Table 2:** Comparison of indications for antenatal corticosteroids administration among groups.

**Variables**	**Delivered <2 d after ACS administration (*n =* 159)**	**Delivered 2–7 d after ACS administration (*n =* 131)**	**Delivered >7 d after ACS administration (*n =* 137)**	**P-value**
Indications for ACS, *n* (%)				**<0.001**
Preterm labor, *n* (%)	2 (1.3)	0	24 (17.5)	
PROM, *n* (%)	13 (8.2)	3 (2.3)	1 (0.7)	
Fetal indications, *n* (%)	17 (10.7)	21 (16.0)	46 (33.6)	
Maternal indications, *n* (%)	127 (79.9)	104 (79.4)	54 (39.4)	
Abnormal vaginal bleeding, *n* (%)	0	2 (1.5)	11 (8.0)	
Asymptomatic changes of the cervix, *n* (%)	0	1 (0.8)	1 (0.7)	

*PROM, premature rupture of membranes; ACS, antenatal corticosteroids. Bold values are statistically significant*.

Multivariate analysis showed that the incidences of RDS/TTN were similar among the 4 groups (Tables 3–5). The associated risk for neonatal hypoglycemia was highest in the delivery with <2 days group (adjusted odds ratio [aOR]: 2.684, 95% confidence interval [CI]: 1.647–4.374 for control group; aOR: 2.827, 95% CI: 1.250–6.392 for delivery 2–7 days group; aOR:2.975, 95% CI: 1.265–6.996 for delivery >7 days group) (Tables 3–5). Of the 159 neonates who were delivered within 2 days of ACS administration, 73 were delivered within 24 h and 86 were delivered between 24 and 48 h after administration. The incidence of RDS/TTN was similar between the 2 groups, while the incidence of hypoglycemia was significantly lower (11 vs. 32.6%; *P* = 0.001) among neonates who were delivered within 24 h of ACS administration than among those who were delivered between 24 and 48 h after administration (Table 6).

**Table 3:** Association between time interval from antenatal corticosteroids administration to delivery and adverse neonatal outcomes.

**Outcome**	**Adjusted OR (95%CI)**	***P*-value**
**RDS (including TTN)**
<2 d after ACS	1.308 (0.410, 4.170)	0.650
2–7 d after ACS	–	0.996
>7 d after ACS	0.943 (0.565, 1.575)	0.823
ACS not administerd	Ref	
**Hypoglycemia**
<2 d after ACS	2.684 (1.647–4.374)	**<0.001**
2–7 d after ACS	1.128 (0.822–1.548)	0.455
>7 d after ACS	0.986 (0.791–1.229)	0.902
ACS not administerd	Ref	

*Adjusted for hypertensive disorders, gestational age, birth weight, small for gestational age. OR, odds ratio; CI, confidence interval; ACS, antenatal corticosteroids; RDS, respiratory distress syndrome; TTN, transient tachypnea of the newborn; Ref, reference. Bold values are statistically significant*.

**Table 4:** Association between time interval from antenatal corticosteroids administration to delivery and adverse neonatal outcomes.

**Outcome**	**Adjusted OR (95%CI)**	***P*-value**
**RDS (including TTN)**
<2 d after ACS	–	0.996
2–7 d after ACS	Ref	
>7 d after ACS	–	0.993
**Hypoglycemia**
<2 d after ACS	2.827 (1.250, 6.392)	**0.013**
2-7 d after ACS	Ref	
>7 d after ACS	0.960 (0.735, 1.253)	0.763

*Adjusted for hypertensive disorders, gestational age, birth weight, small for gestational age, and number of antenatal corticosteroid doses (1~3 or 4). OR, odds ratio; CI, confidence interval; ACS, antenatal corticosteroids; RDS, respiratory distress syndrome; TTN, transient tachypnea of the newborn; Ref, reference. Bold values are statistically significant*.

**Table 5:** Association between time interval from antenatal corticosteroids administration to delivery and adverse neonatal outcomes.

**Outcome**	**Adjusted OR (95%CI)**	***P*-value**
**RDS (including TTN)**
<2 d after ACS	1.727 (0.134, 22.278)	0.675
2–7 d after ACS		0.993
>7 d after ACS	Ref	
**Hypoglycemia**
<2 d after ACS	2.975 (1.265, 6.996)	**0.012**
2–7 d after ACS	1.063 (0.713, 1.586)	0.763
>7 d after ACS	Ref	

*Adjusted for hypertensive disorders, gestational age, birth weight, small for gestational age and number of antenatal corticosteroid doses (1~3 or 4). OR, odds ratio; CI, confidence interval; ACS, antenatal corticosteroids; RDS, respiratory distress syndrome; TTN, transient tachypnea of the newborn; Ref, reference. Bold values are statistically significant*.

**Table 6:** Comparison of neonatal outcomes between newborns delivered within 24 h and those deliveries between 24 and 48 h after early-term antenatal corticosteroid administration.

**Neonatal outcome**	**Delivered <24 h after ACS administration (*n =* 73)**	**Delivered 24–48 h after ACS Administration (*n =* 86)**	***P*-value**
RDS (including TTN), *n* (%)	1 (1.4)	3 (3.5)	0.732
Neonatal hypoglycemia, *n* (%)	8 (11.0)	28 (32.6)	**0.001**

*ACS, antenatal corticosteroids; RDS, respiratory distress syndrome; TTN, transient tachypnea of the newborn. Bold values are statistically significant*.

## Discussion

In this cohort, ACS treatment in ETSCS among women with GDM or DIP, regardless of the time interval to delivery, was not associated with a reduction of RDS/TTN but increased the risk of neonatal hypoglycemia when delivery occurred within 2 days after administration.

Similar to previous studies (14, 22, 23), we found that ACS administration did not reduce respiratory morbidity in early term infants of mothers with any form of diabetes, despite there being some differences in the gestational age of infants and the timing of ACS treatment among studies. In addition, the secondary analysis of a large cohort study found that ACS did not improve neonatal outcomes even when applied to mothers with diabetes between 23^+0^ and 33+6 weeks of gestation (24). While ACS treatment accelerates neonatal pulmonary maturity, it is also well-known that ACS causes maternal hyperglycemia and subsequent fetal hyperinsulinemia, which contribute to delayed pulmonary maturity (25–28). Thus, it is possible that neonates born to women with GDM or DIP do not receive the same benefit from ACS, compared with those of mothers uncomplicated by diabetes, because of the maternal and fetal metabolic side effects of corticosteroids exposure, as well as the underlying glycemic metabolism disorders (24).

We observed an increased likelihood of neonatal hypoglycemia among neonates born within 2 days of ACS administration, which was consistent with findings of other's research (14, 29). Gupta et al. (14) found that ACS administration in ETSCS among women with GDM or DIP is associated with more neonatal hypoglycemia (24.2 vs. 4.4%; aOR, 18.96; 95% CI, 2.18–165.23), although they made the presumption that ACS administered at different time points before delivery would confer equal risk to neonatal hypoglycemia. A recently large cohort study (115/1,248 women with GDM) demonstrated that late preterm infants' risk of hypoglycemia decreased as the time interval from ACS administration to delivery increased, and the risk for hypoglycemia was highest in the delivery of <2 days group (29). Reports have suggested that even mild hypoglycemia which responds to treatment is still associated with poor neurocognitive and developmental outcomes (30). Currently, there is a lack of uniform opinions about whether the use of ACS should extend beyond 34 weeks of gestation among women with diabetes (31). Based on the results of this study, we suggest that ACS may be safely omitted in women with any form of diabetes in pregnancy undergoing ETSCS.

There are several strengths to this study. We evaluated the association between different time intervals from the administration of ACS to delivery and adverse neonatal outcomes in women with any form of diabetes undergoing ETSCS for the first time; a specific and unique population not thoroughly investigated previously. Another strength of this study is our sample size, which included 1,165 early-term neonates from singleton pregnancies exposed to ACS, is among the largest reported in the literature.

This study also has several limitations. This was a single-center retrospective cohort study. The HbA1c in the second or third trimester of some women was unavailable, which is a known risk factor for neonatal hypoglycemia (Table 2) (32), but there was no significant difference among groups based on current data. Although adjusted for in our multivariable analyses, there were some significant differences in the baseline characteristics among the 4 study groups, such as indications for ACS administration, doses of ACS treatment, gestational age, etc., which have been independently associated with adverse neonatal outcomes and may have contributed to our findings. Finally, we do not have data on whether neonates diagnosed with hypoglycemia had any related adverse events, especially impaired neurocognitive development. Therefore, a larger, randomized controlled trial is required to further confirm these findings and assess the long-term impact of hypoglycemia on neonates.

In conclusion, our findings suggest that ACS treatment for women, with any form of diabetes in pregnancy undergoing ETSCS, is not associated with improved neonatal respiratory outcomes, and may increase the incidence of neonatal hypoglycemia, especially when delivery occurred <2 days after ACS treatment. Therefore, we do not recommend administration in this population until further evidence regarding the safety of ACS is available.

## Data Availability Statement

The original contributions presented in the study are included in the article/supplementary material, further inquiries can be directed to the corresponding author.

## Author Contributions

JLi and JZ conceived the idea. JLi and QH analyzed the data. JLi wrote the manuscript. JZ, QH, YD, JLu, HC, and XC reviewed and edited the manuscript.

## Conflict of Interest

The authors declare that the research was conducted in the absence of any commercial or financial relationships that could be construed as a potential conflict of interest.

## Publisher's Note

All claims expressed in this article are solely those of the authors and do not necessarily represent those of their affiliated organizations, or those of the publisher, the editors and the reviewers. Any product that may be evaluated in this article, or claim that may be made by its manufacturer, is not guaranteed or endorsed by the publisher.
